# Insights from precision-cut lung slices—investigating mechanisms and therapeutics for pulmonary hypertension

**DOI:** 10.1186/s12931-025-03290-x

**Published:** 2025-06-21

**Authors:** William R. Studley, Emma Lamanna, Claudia A. Nold-Petry, Cheng Xue Qin, Jane E. Bourke

**Affiliations:** 1 Biomedicine Discovery Institute, Department of Pharmacology, Monash University, Clayton, VIC Australia; 2 Ritchie Centre, Hudson Institute of Medical Research, Clayton, VIC Australia; 3 Department of Paediatrics, Monash University, Melbourne, VIC Australia; 4 Monash Institute of Pharmaceutical Sciences, Monash University, Parkville, VIC Australia; 5 Baker Heart and Diabetes Institute, Melbourne, VIC Australia

**Keywords:** Precision cut lung slices, Pulmonary hypertension, Vasoconstrictor, Vasodilator, Inflammation, Fibrosis, Reactivity

## Abstract

Precision-cut lung slices (PCLS) are gaining traction as a versatile ex vivo tool to study mechanisms and treatments for lung diseases. This preparation, in which the major structural elements of the native lung are preserved, bridges the gap between cell and in vivo models allowing researchers to assess integrated functional responses including smooth muscle reactivity, inflammation and tissue remodelling. To date, the application of PCLS to study outcomes relevant to diseases affecting the pulmonary vasculature, such as pulmonary hypertension, is relatively limited compared to those focussed on chronic airway or interstitial lung diseases.

This review explores the specific technical requirements for the preparation of PCLS with viable, patent pulmonary arteries, and their application for investigation of mechanisms and treatments related to pulmonary hypertension. Studies characterising vascular responses to contractile agonists in PCLS, particularly in the context of disease-relevant stimuli and models are described, as well as the use of PCLS for the identification of novel vasodilators. This article also outlines current research to prolong PCLS viability and provides directions for future PCLS studies to investigate inflammation and vascular remodelling, with a view to identify therapeutics that address the current limitations of dilator-only treatment of pulmonary hypertension.

Overall, the review highlights the importance of PCLS for mechanistic studies and drug development. While PCLS are currently underutilised in the context of pulmonary hypertension, the evidence provided here of the multifaceted functional outcomes that can be investigated using PCLS supports their wider application for understanding disease pathophysiology and validating novel therapeutics.

## Background

Respiratory diseases are among the leading causes of death globally, After heart disease, stroke and cancer [1]. While pulmonary hypertension (PH) is less prevalent than other chronic lung diseases, its incidence is increasing [2]. The various types of PH vary in their underlying causes, with Group 1 pulmonary arterial hypertension (PAH) considered idiopathic, Groups 2, 3 and 4 PH associated with left heart disease, lung diseases and/or hypoxia or with chronic pulmonary artery obstruction respectively and Group 5 PH due to unclear and/or multifactorial mechanisms [3]. All groups are defined by a sustained elevation in mean pulmonary arterial pressure (mPAP), resulting in right ventricular dysfunction [4]. Despite the large heterogeneity in their pathophysiological pathways, progressive remodeling of the distal pulmonary vasculature is a common feature. This review focusses on the preparation and application of precision-cut lung slices (PCLS) to study disease mechanisms and therapeutics related to PH. The focus on studies assessing vascular reactivity is of particular relevance to group 1 PAH, but also to Groups 3 and 4 where pre-capillary PH is also present.

Current therapies for PAH are largely limited to dilators targeting the elevated pulmonary vascular resistance, comprising endothelin receptor antagonists, phosphodiesterase inhibitors, soluble guanylate cyclase inhibitors and prostacyclin receptor agonists [4]. They have limited efficacy in affecting the underlying processes associated with disease progression, so the prognosis for patients remains poor after diagnosis [5].

The identification of more effective treatments requires experimental models to bridge the gap between in vitro cellular studies and in vivo intervention studies. Precision-cut lung slices (PCLS), in which the major structural elements of the native lung are preserved, provide an invaluable preparation for investigating lung physiology and pharmacology in health and disease [6, 7]. PCLS are prepared by slicing lung lobes or resections inflated with low melting point agarose, allowing key pulmonary cells within airways, arteries and parenchyma to be maintained within the native connective tissue [7, 8]. A recent study also described the relatively complex immune compartment in human PCLS, which included monocytes, natural killer and T cells, as well as macrophages [9].

Notably, the presence of patent intrapulmonary airways and arteries in PCLS allows constrictor and dilator responses, and the influence of exposure to disease-relevant stimuli or disease models on their reactivity, to be observed in situ. To date, PCLS have predominantly been utilised for studying airway reactivity with fewer studies on inflammatory responses and tissue remodelling. The potential application of PCLS for the integrated measurement of outcomes relevant to pulmonary hypertension has been largely overlooked, possibly due to the greater challenges in preparing PCLS with intact arteries compared to airways.

Historically, tissue slices from many organs have been used for pharmacology and toxicology studies for decades. However, the preparation of lung slices with preserved structure initially presented challenges compared to the slicing of solid organs, such as the liver and kidney. Several groups successfully prepared hand-cut lung slices (1–2 mm thick) following inflation of the lung with low melting point agarose to support the deflated lung tissue [10, 11]. These slices were then used for toxicological testing [11] and the first demonstration of the contraction of intrapulmonary airways in situ [10]. The use of a vibratome to prepare “precision-cut” lung slices (typically 200–300 μm thick) resulted in improved imaging under phase-contrast and greater methacholine-induced contraction of individual airways [12].

Since this time, the use of PCLS for preclinical and translational research has increased exponentially in laboratories worldwide. The reader is directed to numerous excellent reviews summarising the varied applications of PCLS, encompassing studies extending from animal models to human lung [6, 7, 13–17].

This review focuses on the applications of PCLS for pharmacological studies of the pulmonary vasculature, and their relevance to pulmonary hypertension. It addresses specific methodologies in the preparation and culture of PCLS that enable studies to assess the influence of disease-relevant stimuli/models and to identify novel vasodilators. In addition to describing the advantages and key considerations in using PCLS to characterise vascular reactivity, the review highlights the gaps in current research related to other outcomes that can be measured in PCLS, providing directions for future research.

## Considerations for preparation of PCLS with intact pulmonary arteries

Numerous publications have described the preparation of PCLS from different species in detail [13, 18–21]. While many of the steps in their preparation might be considered standard, considerable variations between methodologies have developed in different laboratories. These variations are often associated with the wider implementation of their use for different readouts, supporting the need for minimum reporting of key details to achieve consensus [8]. Here, the specific additional considerations in preparing PCLS for studies focused on the vasculature are described.

### Standard agarose inflation

In all instances, the preparation of PCLS requires the lung to be first inflated with agarose, due to the soft and pliable nature of lung tissue. Agarose is delivered as a warm liquid, either via the trachea in mice or via large airways in whole lung lobes from larger species. To inflate smaller human lung samples lacking an intact pleura, agarose can be delivered via microinjection directly into alveolar tissue [22, 23]. The inflated sample can then be cooled to solidify the agarose, then cored or cut in uniform blocks before multiple sequential PCLS of uniform size and the required thickness are cut. Agarose-only inflation remains the most commonly used protocol used experimentally, resulting in PCLS containing patent airways. Studies describing the integrated readouts such as inflammatory mediator release, initiation of fibrosis in addition to visualisation of airway contractile responses using PCLS prepared using this approach have been extensively reviewed [6, 13, 14].

### Preserving patent arteries

By comparison, there is a relative lack of studies using PCLS to assess the reactivity of arteries in either mouse or human PCLS. This can be at least partially attributed to the technical challenges of preparing PCLS that possess patent arteries. As the lung is inflated with agarose, the majority of small pulmonary arteries collapse due to their poor tethering to the surrounding tissue [7]. Those that do remain open are usually filled with coagulated blood, precluding contractile responses required for reactivity studies.

Several peer-reviewed scientific video articles and publications have described protocols for the preparation of mouse and human PCLS specifically for vascular studies [7, 18, 20, 24, 25]. One approach to preserve artery structure in mouse PCLS is to avoid the use of inhaled anaesthetics such as isoflurane and halothane for euthanisation, due to their potential effects on vascular tone [26, 27].

Paddenberg et al. achieved promising results by perfusing the pulmonary vasculature with a vasodilator solution, such as sodium nitroprusside, before lung lobe inflation [24]. More robust approaches have been developed whereby the blood in the pulmonary vasculature is replaced by perfusion with either a heparin or gelatin solution before tracheal inflation with agarose [24, 25]. Patent arteries in PCLS responding to a range of constrictors and dilators were obtained after clearing the pulmonary circulation with a buffer containing the anticoagulant heparin, within the range of 250–1000 IU before lung inflation [24, 28]. Perez and Sanderson found that perfusion of a 6% gelatin solution into the right ventricle as part of a two-step process consistently resulted in PCLS with patent viable arteries. Critically, gelatin dissolves upon warming to 37˚C, so it is readily removed from the arteries during culture of PCLS. In these experiments, Ca^2+^-mediated vasoconstriction of intrapulmonary arteries in response to serotonin (5-HT) and KCl was demonstrated [25].

**Fig. 1:**
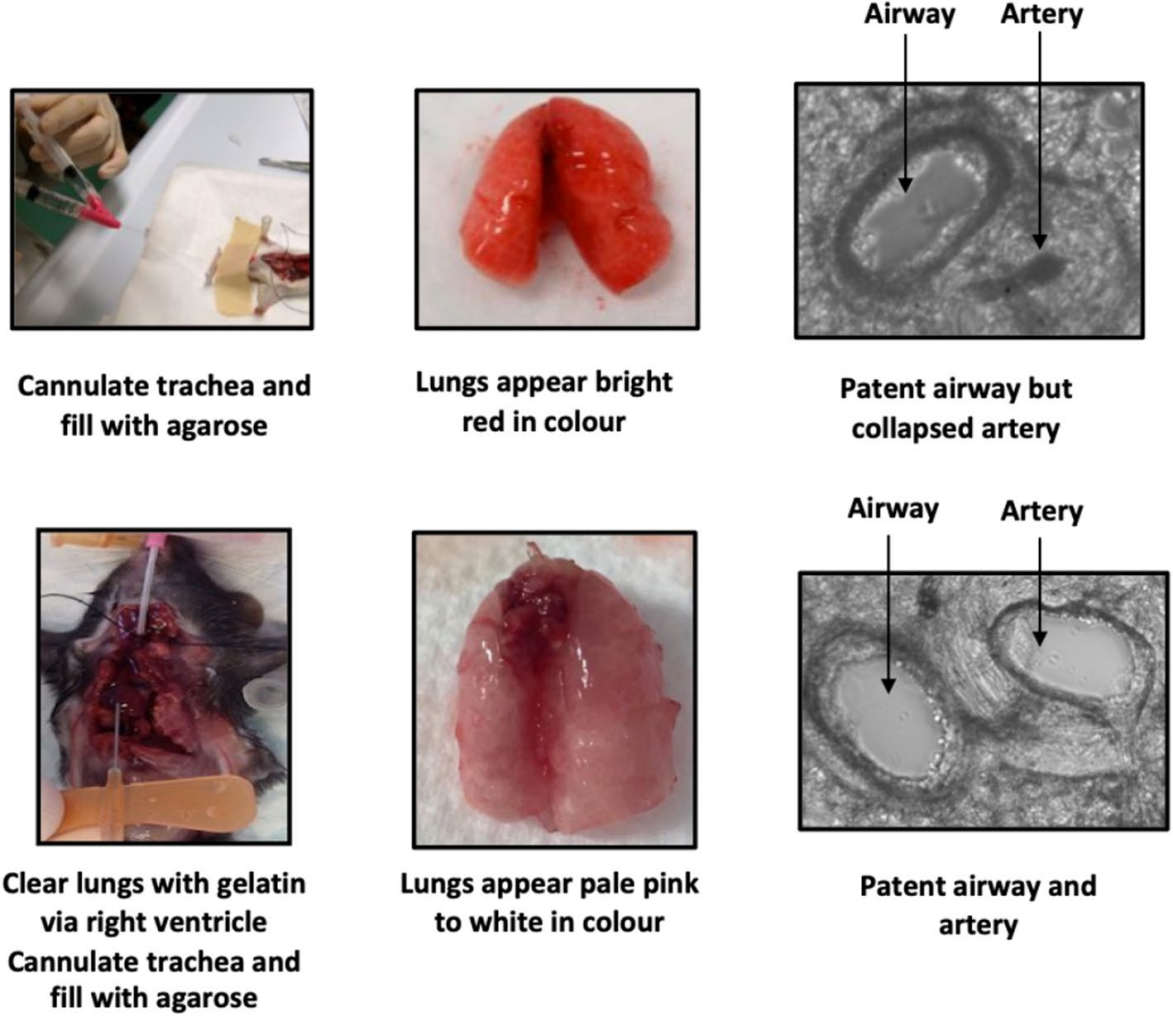
Comparison of preparation of mouse precision-cut lung slices with and without gelatin perfusion. As most methodologies utilising mouse PCLS focus on airway reactivity and/or integrated tissue responses such as inflammation or fibrosis, the most common method of preparation is done so without regard for preserving pulmonary artery structure. With agarose inflation of the airways alone, most pulmonary arteries are either collapsed, or filled with coagulated blood, which restricts vasoconstriction (upper panels). Perfusion with a heparin or gelatin solution via the right ventricle clears the vasculature, as shown by the pale pink appearance of the inflated lung and increases the preservation of artery structure and patency (lower panels). While this approach can be used in intact lobes, smaller resection samples may lack readily accessible pulmonary airways or arteries to supply the lung segment, and/or an intact pleura required to retain agarose and gelatin

The effect of gelatin infusion of the vasculature in preserving the patency of pulmonary arteries in mouse PCLS (in addition to airways) is clearly illustrated in Fig. 1. There have been no direct comparisons between the various methods of PCLS preparation regarding their efficacy at clearing blood, maintaining tissue structure with slicing, and preserving vascular reactivity. Of note, functional intact arteries have been identified in human PCLS using standard agarose inflation protocols in the absence of heparin or gelatin infusion, suggesting that the tethering of human pulmonary arteries to the surrounding parenchyma may be more robust than in the mouse [25, 29–34].

### PCLS culture

As mentioned earlier, multiple PCLS can be obtained from a single lobe of mouse lung, and potentially hundreds of PCLS from a single lobe of larger species. Irrespective of the readout to be measured, it is recommended that PCLS are washed and incubated for up to 24 h before commencing experiments, allowing for recovery of metabolic activity following tissue preparation [35]. The recent ATS Workshop Report on PCLS highlighted inconsistencies between research groups in reporting conditions for both the preparation and culture of PCLS, often lacking sufficient detail to permit studies to be reproduced or extended by other investigators [8]. The marked variability in culture conditions for related studies is highlighted later in this review under *Vasodilation studies using PCLS* (Table 1). Furthermore, efforts being made to prolong the viability of PCLS and validate the retention of functional responses including contraction after cryopreservation are detailed under *Knowledge Gaps and Future Directions*.

**Table 1:** Application of precision-cut lung slices from different species and disease models to assess vasodilation of intrapulmonary arteries to various compounds

Drug class	Vasodilator	Pre-contraction	PCLS source *(model/disease)*	Media	Ref
Endothelin Receptor Antagonist	Bosentan	ET-1	Human *(non-PH)*	DMEM (10% FCS, 1% amphotericin B, 2% Pen/Strep)	[30]
Phospho-diesterase inhibitor *(target enzyme)*	Sildenafil *(PDE5)*	5-HT	Mouse *(naïve)*	DMEM (1% Pen/Strep)	[36]
	Milrinone *(PDE3)*	BP0104 (ETA agonist)	Human (*non-PH)* Guinea Pig *(naïve)*	No information	[37]
	Papaverine *(PDE10 A)*	5-HT	Human *(IPF* ± *PH)*	RPMI 1640 (100 units/ml Pen, 0.1 mg/ml Strep, 4 mM L-glutamine)	[29]
Soluble Guanylate Cyclase Activator	Riociguat	5-HT	Mouse *(naïve)*	DMEM (1% Pen/Strep)	[36]
Prostacyclin Analogue	Iloprost	5-HT	Mouse *(naïve)*	DMEM (1% Pen/Strep)	[36]
NO Donor	Sodium Nitroprusside	U46619	Mouse *(newborn, naïve)*	DMEM (100 units/ml Pen, 0.1 mg/ml Strep, 4 mM L-glutamine, 2.5 µg/mL amphotericin B)	[38]
	NOC-5	ET-1	Mouse *(PH: OVA-HX)*	DMEM/F12	[39]
Ca^2+^ Sensitiser	Levosimendan	ET-1	Human (*non-PH*)	No information	[33]
		BP0104	Guinea Pig *(naïve)*	No information	[40]
GABA-R activator	Argon	ET-1	Human (*non-PH*) Rat (*naïve)*	No information	[34]
Muscarinic receptor agonist	ACh	ET-1, U46619	Mouse *(newborn, naïve)*	DMEM (100 units/ml Pen, 0.1 mg/ml Strep, 4 mM L-glutamine, 2.5 µg/mL amphotericin B)	[38]
		U46619	Guinea Pig *(naïve)*	HEPES/BCM	[41, 42]
		U46619	Rat *(naïve)*	DMEM (100 units/ml Pen, 0.1 mg/ml Strep, 4 mM L-glutamine, 2.5 µg/mL amphotericin B)	[43]
		5-HT	Guinea Pig *(POPV)*	BCM	[44]
Tyrosine Kinase Inhibitor	Imatinib	ET-1	Human* (non-PH) *Mouse *(naïve)*	No information	[32]
	Imatinib	ET-1	Guinea Pig *(naïve)*	No information	[45]
	Nilotinib	ET-1	Human *(non-PH)*	No information	[32]
JAK2 inhibitor	JSI-124	5-HT	Human *(IPF* ± *PH)*	RPMI 1640 (100 units/ml Pen, 0.1 mg/ml Strep, 4 mM L-glutamine	[29]
Formyl Peptide Receptor Agonist	Compound 17b, Compound 43	5-HT	Mouse *(naïve* ± *TNF-α *in vitro*)*	DMEM (1% Pen/Strep)	[36]

*5-HT* serotonin, *ACh* acetylcholine, *BP0104* experimental endothelin receptor agonist, *BCM* bicarbonate-buffered culture medium, *DMEM* Dulbecco’s Modified Eagle Medium, *ET-1* endothelin-1, *GABA-R* gamma-aminobutyric acid receptor, *IPF* idiopathic pulmonary fibrosis, *JAK2* Janus kinase 2, *JSI-124* cucurbitacin I, *NO* nitric oxide, *NOC-5* 3-[2-hydroxy-1-(1-methylethyl)−2-nitrosohydrazinyl]−1-propanamine, *OVA-HX* ovalbumin-hypoxia, *pen* penicillin, *PH* pulmonary hypertension, *strep* streptomycin, *U46619* 9,11-Dideoxy-9α,11α-methanoepoxyprostaglandin F2α, thromboxane A2 mimetic

## Advantages and considerations in the use of PCLS to study vascular responses

### Assessment of pre-acinar arteries in situ

Contraction and relaxation responses of intrapulmonary arteries can be assessed in PCLS, providing a platform to investigate disease mechanisms and potential therapies, particularly related to pulmonary hypertension. Critically, the small, muscular, pre-acinar arteries present in PCLS, ranging from 250-50um diameter, are key contributors to vasoreactivity-mediated changes in mPAP [46] and are the major site of vascular remodelling in PAH patients [47]. These arteries are distal to the main pulmonary arteries, and to the branching elastic pulmonary arteries in the pulmonary trunk that form bronchovascular bundles with the bronchial tubes. These smaller muscular pulmonary arteries branch further into intra-acinar arterioles and into pulmonary capillaries that are largely devoid of smooth muscle and surround the alveoli at sites of gas exchange.

Prior to the development of PCLS, in vitro pharmacological assessment of the reactivity of these muscular, pre-acinar arteries presented significant challenges since they are too small to be easily dissected as intact rings for traditional myography. The majority of studies characterising pulmonary arteries have used readily accessible larger pulmonary arteries (> 1 mm), mounted in a myograph or organ bath under isometric conditions. In studies using mouse or rat models, reactivity has most commonly been assessed using the isolated main pulmonary artery, first or second order branches [48–50].

The preparation of PCLS with intact intrapulmonary arteries was initially reported in small animal species [12, 28] but has more recently been established using human lung [30]. Clearly, PCLS provide a more physiologically relevant microenvironment than isolated arteries in an organ bath, as the intrapulmonary arteries remain tethered to the surrounding lung parenchyma and in close proximity to airways in situ as in the intact lung [25, 51]. Functional responses of intact arteries that are not possible in single cell culture systems are maintained, with the added benefit of preservation of complex cell-to-cell and cell-to-matrix relationships that are lacking in isolated artery rings. With this technique, responses to constrictors or dilators can be quantified via imaging software as changes in lumen area under phase-contrast microscopy. The PCLS technique also offers the unique capacity for simultaneous assessment of adjacent intrapulmonary airways and arteries, although this has only rarely been performed [12, 25, 51, 52].

### Artery selection

A key consideration in the use of PCLS is the criteria that should be applied for selection of an artery for assessment. Depending on the location of an individual PCLS within the lung, the number of arteries will vary, so visual inspection is required to identify an artery adjacent to an airway with beating cilia as an indicator of slice viability. To exclude the possibility of measuring reactivity of a vein rather than an artery, particularly from human samples, Reig et al. also reports histological staining of PCLS after experiments with Elastica van Giessen stain [32].

### Multiple PCLS per lung sample

**Fig. 2:**
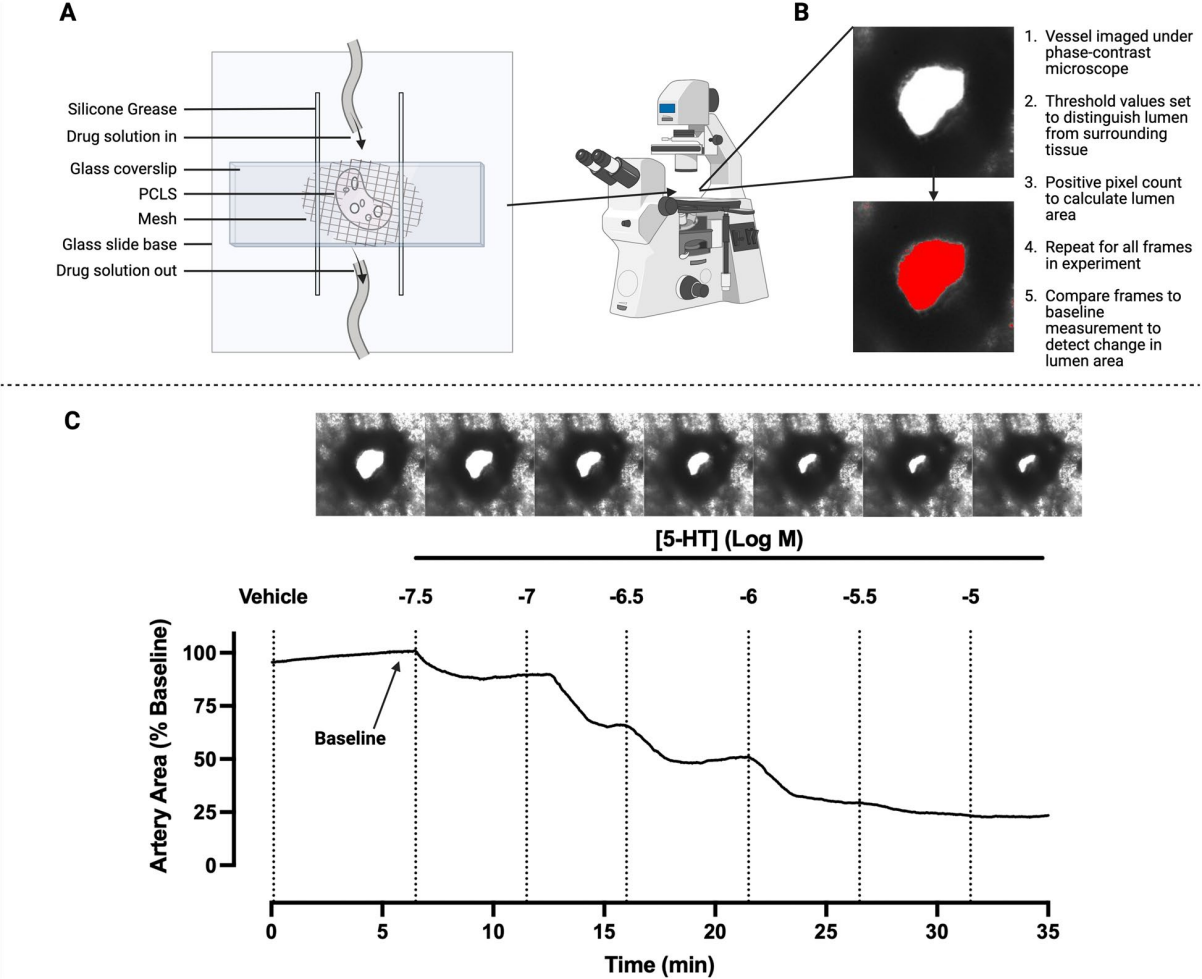
Representative experimental set-up, with images and trace for concentration-dependent pulmonary artery contraction to 5-HT in human PCLS. **A** Diagram of a custom-made perfusion chamber for PCLS reactivity studies. A single PCLS is mounted on a glass coverslip supported in a perspex frame. The PLCS is held in place under a piece of plastic mesh with a small hole positioned over the vessel/airway of interest. Silicone grease is laid in parallel lines over the mesh, and another coverslip is paced on top to create a chamber with enclosed sides and two open ends. A constant-perfusion drug delivery system is established, introducing buffers or drugs of interest to the chamber under gravity, and removing them under vacuum. This chamber is positioned over a phase contrast microscope and imaged over time. **B** Analysis of captured images used to calculate changes in artery lumen area. **C** Human PCLS from healthy margin of lung resection specimen was incubated overnight after preparation, then mounted in a perfusion chamber and exposed to 1X HBSS for 5 min to establish baseline area. Increasing concentrations of 5-HT, in half log molar increments, were perfused through the chamber every 5 min. Images were captured every 2 s, with a total of 1050 captured frames. Lumen area was analysed from TIFF files using ImageJ software, using method in (**B**). Artery area is normalised to the baseline artery lumen area. Representative images show the last frame of each treatment. 5-HT: 5-hydroxytryptamine

Many experimental protocols using PCLS measure concentration–response curves to a given drug, as shown for 5-HT-mediated contraction in human PCLS (Fig. 2). Critically, the PCLS technique provides a high yield of preparations from each lung sample. Approximately 20 individual PCLS can be generated from a single mouse lobe, compared to only a few airway or artery ring preparations from a single experimental animal for traditional organ bath in vitro experiments. In human samples, hundreds of PCLS can be made from a single lobe, or even a relatively small resection. The PCLS technique therefore offers potential advantages compared to in vivo studies, including reduction and replacement of animal use, and opportunities for the use of donor or explant human tissue in place of animal tissue.

### Experimental considerations for treatment and analyses

In our laboratory, an individual PCLS is mounted in a chamber and the selected artery visualized for exposure to a drug under either static or perfused conditions, as shown (Fig. 2). Under static conditions, drugs are added to a fixed volume of buffer within the PCLS chamber, thus maintaining the potential contribution of endogenous mediators such as NO released from the endothelium to the overall response. Under perfused conditions, any secreted mediators would be continuously removed, establishing the direct effect of the drug. Under these circumstances, a temporary halt to the perfusion may still allow the involvement of endogenous mediators in mediating or modulating any response to an exogenous drug to be identified.

Capturing images at regular intervals to follow the time-course of response can be informative (Fig. 2C). In the absence of continuous readout of artery diameter, it is also important to ensure that sufficient time is allowed for plateau responses to a given concentration to be achieved to ensure accurate estimation of drug potency and efficacy in vitro. However, it should be acknowledged that these parameters may not reflect responses to inhaled or systemic drug delivery and, thus, translation of effective concentrations in PCLS to dosing in an in vivo model remains challenging [53].

Within a given study, consistency of artery size is also important, as relatively greater contractile responses and significantly lower nitric oxide (NO)-induced vasodilation have been demonstrated in proximal pre-acinar arteries (lobular to subsegmental arteries, diameter > 50 µm) compared to distal intra-acinar arteries (microvessels embedded in alveoli, diameter mostly < 40 µm) in mouse PCLS [39]. However, it should be noted that since many sequential PCLS can be generated from an inflated lung lobe or segment, multiple pharmacological agents can be assessed in adjacent slices containing sections from the same artery, and comparisons can be made between matched treated and untreated slices from the same lung sample. To correct for small differences between artery sizes either from the same or separate samples, changes in area in response to constrictor and dilator agents are generally normalised to the initial area in the absence of stimulus.

Overall, the PCLS technique provides an important bridge for translation from cell-based high-throughput assays to studies in the intact animal, and can be used for physiological, pharmacological and toxicological studies [16]. Their application can be extended for comparisons both within and between ex *vivo* and in vivo models relevant to pulmonary vascular diseases.

As detailed below, PCLS have been prepared from naïve animals or healthy margins of human resection specimens and treated ex vivo to explore the acute effects of disease-relevant stimuli such as inflammatory cytokines or LPS on pulmonary artery constrictor and dilator responses [30, 31, 36, 52]. PCLS from diseased human donors [29] or from in vivo rat or mouse models of disease including ARDS, BPD and PH [13, 35, 39, 52, 54–58] have been used to test the effects in vivo or in vitro treatments on disease-induced changes measured ex vivo. These approaches can inform in vivo studies in the same species, as the initial responses of resident cells can be measured without the complicating influence of systemic immune or physiological responses [59].

## Assessment of intrapulmonary artery reactivity in PLCS

### Artery contraction in PCLS

#### Naïve PCLS

Contractile responses of intrapulmonary arteries have been established in PCLS from mouse, rat, guinea pig and human lungs to a range of known endogenous mediators, including ET-1, 5-HT, the stable thromboxane mimetic U46619, angiotensin-II, histamine, PDGF and adrenergic agonists [25, 43, 60–64]. Mechanistically, Perez and Sanderson showed that in both airways and arteries, contraction to 5-HT and ET-1 in PCLS is modulated by the sensitivity to, and frequency of, Ca^2+^ oscillations in artery smooth muscle cells [25, 51, 60]. As described previously, Bai et al. recently delineated differences in contractile responses of intra-acinar and pre-acinar arteries to ET-1 in mouse PCLS, consistent with previously reported regional differences airway contractile responses to the broncho-constrictor methacholine [39, 65].

Hypoxic pulmonary vasoconstriction, a consequence of ventilation-perfusion mismatching in vivo, has been established in mouse, rat and human PCLS ex vivo [24, 28, 34, 66]. A requirement for precontraction of pulmonary arteries to observe the response to hypoxia appears to be species-dependent as it was only required in rat PCLS [67]. The use of PCLS has provided a mechanism underlying this response. Super-fusion with hypoxic media released reactive oxygen species (ROS) and increased calcium signalling [66], and inhibition of ROS signalling effectively blocked the vasoconstrictor response to hypoxia in mouse PCLS [28].

#### Ex vivo models

Several studies have exposed PCLS to inflammatory stimuli relevant to pulmonary vascular disease, establishing ex vivo models to assess potential influences on contraction to agonists known to be important in PH. For example, levels of interleukin 11 (IL-11) and its soluble receptor IL-11Rα were demonstrated to be elevated in pulmonary arteries from PH patients [31]. In showing that treatment of non-diseased human PCLS with either the cytokine or its receptor sensitised pulmonary arteries to ET-mediated contraction, the utility of PCLS to implicate a specific pathway to PH pathophysiology was achieved [31].

#### From in vivo models

PCLS have been prepared from a mouse model of acute respiratory distress syndrome (ARDS), in which mice were administered lipopolysaccharide (LPS) over 4 days to mimic the response to bacterial infection in vivo [52]. The increased contraction of intrapulmonary arteries to ET-1 and U46619 in PCLS was also evident following acute treatment with LPS ex vivo and replicated in PCLS treated with TNF-α alone, implicating this cytokine as a driver of the altered contraction. These combined findings clearly demonstrate that an intact immune response is not required to elicit changes in the contractile responses of vascular smooth muscle cells [52].

PCLS have also been prepared from other models in which key features of pulmonary vascular diseases have been established in vivo. Contraction and dilation of pulmonary arteries and veins have also been measured in PCLS from a guinea pig model of post-obstructive pulmonary vasculopathy (POPV), demonstrating increased contraction to 5-HT and histamine while NO-mediated relaxation was maintained [44].

PH secondary to bronchopulmonary dysplasia (BPD-PH) is a major complication of this disease of prematurity, predictive of early mortality [68]. Longer term respiratory symptoms and impairment in lung function have also been reported in survivors [69]. A double-hit model of bronchopulmonary dysplasia (BPD) combining in utero LPS-induced inflammation and postnatal exposure of pups to 65% O_2_ rich gas for 28 days (hyperoxia) has been established, with exposure to room air serving as the control [70]. Echocardiography established the development of PH in the hyperoxic mice, associated with persistent inflammation, perivascular fibrosis and vascular rarefaction in the absence of increased contraction of intrapulmonary arteries to either endothelin-1 or U46619 in PCLS [57]. Interestingly, airway hyperresponsiveness to MCh was observed in PCLS from this model [71], demonstrating the unique potential for PCLS to identify differential effects of a disease model on airway versus artery reactivity.

Studies using PCLS from models of PH to investigate the potential contribution of altered reactivity of intrapulmonary resistance arteries to disease remain limited. A single study in a rat monocrotaline model established an increase in maximal contraction to ET-1 in arteries, but not airways, consistent with the known contribution of this potent vasoconstrictor to PH [72]. More recently, Bai et al. utilised PCLS from a 4-week ovalbumin (OVA)/hypoxia (HX) model of PH [39]. Despite evidence of vessel wall hypertrophy in both pre-acinar and intra-acinar arteries, only the former became hypercontractile to ET-1 following OVA-HX, accompanied by a localised reduction in NO-mediated relaxation [39]. This latter paper highlights the insights that can be obtained by integrating an in vivo model with relevant PCLS readouts to elucidate region-specific effects on both constrictor and dilator responses throughout the lung.

To date, only a single study has addressed the influence of disease on contractile responses of intrapulmonary arteries in human PCLS. IPF is known to be associated with the development of Group 3 PH characterised by reduced lung compliance due to interstitial fibrosis. Vasoconstriction to 5-HT was significantly lower in PCLS from IPF patients with compared to those prepared from non-disease lung [29]. This suggests that the measurement of vascular reactivity in situ in PCLS has allowed the important role of interstitial compliance in regulating vascular responses to be confirmed.

These combined studies establish that vascular contractile responses in PCLS can be altered by treatment in vitro with disease-relevant stimuli and maintained ex vivo when prepared from models that mimic characteristics of disease. In addition to providing mechanistic insights, these approaches establish conditions to assess novel interventions to oppose excessive vasoconstriction evident in many pulmonary vascular diseases.

### Vasodilation studies using PCLS

For diseases that affect the pulmonary vasculature, PCLS remain an underutilised tool to bridge preclinical and clinical studies, particularly in the identification of novel vasodilators.

Studies that have investigated vasodilator responses mediated by various mechanisms in PCLS from different species are shown, typically assessed after precontraction with contractile agonists implicated in diseases such as PH, including ET-1, U46619 or 5-HT (Table 1). There is marked variability or lack of reporting of media and supplements in these studies. PCLS were mostly cultured in DMEM supplemented with pen/strep, with two studies using BCM or RPMI 1640, and one using HEPES alone. Details of supplementation with serum were limited to a single study, while PCLS in two studies were treated with an antimycotic in addition to antibiotics. Notably, six studies failed to report culture conditions. Future studies should provide the recommended minimum reporting standards of the ATS Report [8] including culture media and supplements used, frequency of media changes, as well as time before treatment, duration of incubation and information on experimental design and technical replicates to enable results to be compared or replicated between studies [36].

Across several studies, vasodilators in clinical use for PAH, including sildenafil, iloprost, riociguat and bosentan have consistently shown efficacy in both mouse and human PCLS, [29, 36]. This validates PCLS as a pre-clinical model for the testing of novel vasodilators, providing new candidates for the treatment of pre-capillary or even all groups of PH. Current therapies oppose ET-1-mediated contraction (ET receptor antagonists) or mediate relaxation via activation of NO/sGC/cGMP signalling (NO, riociguat), AC/cAMP signalling (prostacyclin analogues) or inhibition of cGMP breakdown (PDE-5 inhibitors). Drugs in all of these classes have been shown to dilate pulmonary arteries in PLCS from various species, including human. While these acutely relieve mPAP and significantly improve clinical outcomes, they have not been shown to affect the underlying vascular remodelling or prevent disease progression. Of note, PCLS can be used for integrated assessment of inflammatory and dilator responses that could provide additional benefit for alternative therapies over current dilators.

ET-1 is a key driver of PH, due to its actions as a potent vasoconstrictor as well as driving remodelling though its proliferative effects on vascular smooth muscle. Of note, in human PCLS prepared from explant tissue, the increased contraction of intrapulmonary arteries to ET-1 induced by in vitro cigarette smoke extract exposure was prevented by bosentan, a dual ETA/B receptor antagonist [30].

The relative potency of the widely used PDE-5 inhibitor sildenafil, more recently approved sGC activator riociguat and the prostacyclin analogue iloprost have been directly compared in naïve mouse PCLS with 5-HT constricted arteries [36]. In this setting it was shown that all dilators achieved an equal efficacy, but potency of iloprost was far greater than sildenafil and riociguat.

Other phosphodiesterase inhibitors milrinone (PDE-3) and papaverine (PDE-10A) have been shown to elicit relaxation of pulmonary arteries pre-constricted with an ETA receptor agonist BP0104 or 5-HT, respectively [29, 37]. Upstream of these dilators, acetylcholine has been shown to dilate pulmonary arteries in PCLS generated from guinea pigs and rats demonstrating the preservation of endothelium-dependent dilation via release of NO in these preparations [38, 42, 43]. The NO donors sodium nitroprusside and NOC-5 also dilate pulmonary arteries in PCLS prepared from both naive mice [38] and mice with OVA-HX-induced PH despite their increased reactivity to ET-1 [39].

Many of the PCLS studies included in Table 1 involved testing compounds that target pathways that differ from current PH therapies (endothelin receptor antagonists, phosphodiesterase inhibitors, soluble guanylate cyclase inhibitors and prostacyclin receptor agonists). The Ca^2+^ sensitiser levosimendan promotes the dilation of pulmonary arteries and veins in PCLS generated from both guinea pig and human donors [33, 40]. Argon was found to elicit slowly developing relaxation of pulmonary arteries precontracted with ET-1 over 24-h via activation of GABA-R [34]. Argon has shown promise as a neuroprotective agent for patients suffering primary neurological disorder or cardiac arrest/surgery, who are at risk of cardiovascular disorders such as PH. On the basis of their positive findings in rat and human PCLS, the authors of this study suggest a potential role of argon in managing PH in such patients.

The tyrosine kinase inhibitor (TKI) imatinib progressed to clinical trials for PH [73] on the basis of reversal of PDGF-mediated vascular remodelling in a rat MCT model [74] and additional vasodilator properties, as shown in guinea pig and human PCLS [32, 45]. While these combined properties provide an appealing approach to PH treatment, imatinib was shown to have significant pulmonary toxicity [73]. The dilator responses to another TKI nilotinib, which inhibits proliferation of pulmonary artery smooth muscle cells in vitro [74], were investigated as a potential alternative to imatinib [32]. Both TKI inhibitors inhibited pulmonary artery contraction in human PCLS, mediated via relaxation via K^+^ channel activation and generation of cAMP. Further implicating inhibition of tyrosine kinase as a therapeutic approach to oppose vascular contraction, JAK2 inhibitor JSI-124 induced artery relaxation in PCLS from patients with IPF, though in patients with PH secondary to IPF, the relaxant effect was significantly inhibited [29].

As noted earlier, studies demonstrating the efficacy of novel therapies in PCLS only rarely make directly comparisons with approved drugs. Formyl peptide receptors (FPRs) are a class of G-protein coupled receptors (GPCRs) expressed in vascular smooth muscle that may be a novel therapeutic target for PH. The FPR agonist Compound 17b (Cmpd17b) was shown to elicit vasodilation of mouse aorta and reduce vascular remodelling in models of cardiovascular pathologies including myocardial infarction and diabetes [55, 56]. Studley et al. recently provided the first comparison of FPR agonists with current therapies for PH, using mouse PCLS [36]. Both Cmpd17b and Compound 43 (Cmpd43) showed comparable potency and efficacy to riociguat and sildenafil, with similar efficacy but lower potency than iloprost.

PCLS can be treated with inflammatory stimuli as a simplified model for directly assessing inhibitory effects of agents in the absence of an immune cell supply. Notably, vasodilation to Cmpd17b was maintained in PCLS treated with LPS or TNF-α, while the efficacy of iloprost and sildenafil were significantly reduced [36]. Cmpd17b also significantly inhibited TNF-α-mediated IL-6 release from mouse PCLS in vitro, where sildenafil and iloprost did not [36]. These combined findings highlight the potential dual vasodilator and anti-inflammatory actions of Cmpd17b. Further studies to establish FPR agonist efficacy in PCLS from PH models and human PCLS are required. Evidence of potential additional anti-remodelling properties could establish FPR agonists as unique therapeutics targeting both the increased PAP and underlying pathophysiology driving PH progression.

## Knowledge gaps and future directions

### Extending viability for reactivity and other readouts using PCLS studies in vitro

Clearly, the PCLS technique offers multiple benefits for the integrated assessment of intrapulmonary artery (and airway) reactivity as well as acute changes induced in response to disease-relevant stimuli ex vivo. However, the chronic effects of these stimuli are more challenging to assess. Currently, there is uncertainty surrounding the preservation of both contractile responses of arteries and the inflammatory responses that might drive their altered reactivity with longer-term culture of PCLS.

To date, the majority of studies have been performed using PCLS cultured in serum-free media to avoid potential effects of growth factors on responsiveness. Under these conditions, human PCLS airways remain viable and display methacholine-induced bronchoconstriction up to 15 days in culture, but their sensitivity steadily decreases over this time [54]. Supplementation of media with insulin preserves airway smooth muscle contraction in mouse PCLS for up to 2 weeks [75] by protecting against a loss of smooth muscle myosin heavy chain expression in the airways, however it is not known if this also applies for pulmonary artery smooth muscle.

Degradation of the pulmonary vascular endothelium of human PCLS occurs within 5 days in culture and at a faster rate than the airway epithelium [76], which could affect endothelial-dependent dilator responses, such as relaxation to ACh. This loss of endothelial integrity is reported to be exacerbated in fibrotic lung explants, such as those that might come from patients with diseases such as PH or IPF [76]. Live cell imaging of PCLS has shown ongoing interactions between dendritic and T cells in slices prepared from mice following treatment with OVA/LPS in vivo [77]. A separate study showing moderate infiltration of immune cells into areas of infection in PCLS from mice treated ex vivo with *M. tuberculosis* bacteria [78] also suggests that immunological responses are retained in PCLS for at least some time. However, more systematic analysis of changes in viability of different cell populations, differential preservation of cell functions and cell–cell interactions over time have yet to be completed.

Even with daily changes of serum-free media, inflammatory responses to treatment with LPS were reduced over time [35]. As an alternative approach, cold storage of rat PCLS in DMEM/F-12 solutions enriched with potassium chloride and lactobionate resulted in better preservation of CD31 +, CD45 +, CD90 +, and EpCAM + cells, prolonging responses to inflammatory stimulation compared to standard DMEM/F-12 [79].

To extend survival time in culture, several studies have used media supplemented with fetal calf serum (FCS). It was shown that mouse PCLS remained viable for 5 days with no change in live/dead cell staining and maintained secretion of surfactant protein C (SP-C), a measure of alveolar type 2 cell function [80]. Although these outcomes were reduced by day 7, live cell imaging showed that bronchial epithelial cells still showed sustained ciliary activity. Human PCLS cultured in media supplemented with FCS have also been shown to remain viable for up to 5 days with no reduction in mitochondrial activity [81] and for two weeks with no change in tissue viability, metabolic activity or structural integrity [35]. Assessment of the preservation of pulmonary artery reactivity in PCLS with FCS supplementation in extended culture remains a priority for future studies.

### Biobanking and cryopreservation of PCLS

Translation of preclinical findings is likely to be accelerated with validation of findings in human lung, particularly PCLS prepared from patients with the disease of interest. However, many researchers are unable to readily access human lung tissue, or the availability of samples is low and/or unpredictable. Cryopreservation of the multiple PCLS that can be prepared from a single lung sample could provide opportunities to maximise the experimental outcomes that can be obtained and allow for the banking of tissue for later use without depending on maintaining prolonged viability in culture.

To date, individual PCLS have been frozen in dimethyl sulfoxide (DMSO), stored and thawed for study after extended time points. The viability of mouse and rat slices thawed 2–3 days after cryopreservation has been confirmed through measurements of metabolic activity, cell viability and mitochondrial integrity [82]. Additionally, thawed mouse PCLS showed similar metabolic and bronchial reactivity measures to fresh PCLS [83]. More recently, both viability and function of key immune cells, Ca^2+^-dependent mechanisms of airway contraction to MCh and histamine and airway relaxation to bitter taste receptor agonists were shown to be preserved in human PCLS following cryopreservation [84]. A critical knowledge gap remains in the assessment of pulmonary artery reactivity post-cryopreservation.

### Assessment of contributors to tissue remodelling in PCLS

Current therapies for pulmonary vascular diseases such as PH target dysregulated vasoconstriction effectively but fail to impact on underlying structural changes. The unmet need is for therapies whose primary function is to inhibit disease progression, or even reverse the established remodelling evident in a range of vasculopathies, as evidenced by increased muscularisation and fibrosis surrounding pulmonary arteries [85]. PCLS are now providing an invaluable preclinical methodology for assessment of remodelling readouts, that could also be integrated with assessment of reactivity.

#### Proliferation and alveologenesis

Opportunities to perform experiments using PCLS prepared from human explant tissue in which pulmonary artery remodelling is established is limited. Recently, the increase in arterial media thickening in human PCLS in response to treatment with ET-1, PDGF-BB and FGF-2, was inhibited by BMS-303141, an ATP citrate lyase inhibitor, as determined by elastica van Gieson staining [86]. Instead of looking at remodelling directly, researchers can also assess changes in expression of remodelling markers. In human PCLS from COPD patients, treatment with fibroblast growth factor 10 (FGF10) increased cellular proliferation, as characterised by number of BrdU + stained cells, though no proliferating cells were found in the tunica media of the pulmonary arteries [87].

In a novel approach, timelapse imaging of mouse PCLS has captured specific cell behaviours associated with ex vivo septation, epithelial cell migration, and hollowing, consistent with alveologenesis. This potential application of PCLS has been largely ignored in the literature, but these findings suggest that in addition to modelling disease processes, they could be used to assess potential treatments to repair and regenerate damaged lung tissue [88].

#### Apoptosis and vascular pruning

Apoptosis of endothelial cells in the pulmonary vasculature may play a central role in the initiation and progression of PAH [89]. In a cigarette smoke extract model of COPD in human PCLS, the extract increased expression of apoptosis marker propidium iodide, and sensitised the tissue to carcinoembryonic antigen cell adhesion molecule 6-mediated expression of this marker [90].

Another important aspect of PH is vascular pruning, defined as the progressive thickening and termination of small pulmonary arterioles and associated with significant increases in pulmonary vascular resistance COPD-associated PH (92). Vascular pruning, as detected by CT scan, is also significant predictor of pulmonary vascular remodelling (93), so could be incorporated into preclinical models. Bui et al. identified vascular pruning by micro-CT of the whole lung in a mouse model of bronchopulmonary dysplasia in newborns, though this was not correlated with changes in either intimal or medial thickening or increased contractile responses of intrapulmonary arteries remaining in PCLS (49).

#### Fibrosis and inflammation

As a model of generalised parenchymal fibrosis in IPF, PCLS have been treated with disease-relevant inflammatory and fibrotic factors in a fibrotic cocktail (FC) comprising TNF-α, LPA, PDGF and TGF-β [81] to induce collagen deposition ex vivo over 5 days. Additionally, this model verified RNA and protein expression of pro-inflammatory and alveolar reprogramming markers characteristic of IPF. In related studies, FC treatment of PCLS was associated with increased proportions of fibrotic fibroblasts as well as increased α-smooth muscle actin expression and endothelial-mesenchymal transition (EndMT), providing evidence of potential acute vascular remodelling [8, 91, 92]. However, in human PCLS cultured for up to two weeks, TGF-β treatment induced elevated expression of fibrotic markers, despite no changes in collagen deposition within the lung tissue ex vivo [93]. The authors explain this by describing an increase in ECM degradative inflammatory activity, characterised by increased expression of various MMPs. TGF-β-mediated collagen deposition was restored with the addition of MMP inhibitors. These conditions may not be present under normal physiological conditions, perhaps limiting the use of PCLS as a physiologically relevant preparation.

Similar multifactorial models could be adapted to a pulmonary vascular disease such as PH, to provide a relatively high throughput and scalable model for drug development. PH is characterised by elevated expression of most of the cocktail components (i.e. TNF-α, TGF-β, PDGF), while the addition of PH-related components, such as ET-1 or IL-1β, could increase disease specificity. Anti-inflammatory effects of novel drugs compared to current therapies could be assessed in this more disease-relevant setting, extending findings obtained for FPR agonists compared to iloprost and sildenafil in mouse PCLS treated with TNF-α alone [36]. If structural changes such as PDGF-induced smooth muscle proliferation could be established in addition to fibrosis, then the comparative effect of drugs on progression of remodelling could be investigated. Critically, this approach would allow for the comparative assessment of novel dilators with current therapies in inflamed, remodelled arteries in situ.

#### Reversal of remodelling

If stable changes in vascular structure consistent with pulmonary vascular diseases can be achieved with longer-term culture of PCLS, this approach could be extended to reversal studies. Alternatively, samples from animal models of PAH, such as the Sugen/Hypoxia or MCT models, or from PH patients with established vascular remodelling would provide an even more clinically relevant integrated platform. Promising preliminary data has been reported for the novel PDGFR/CSF1R/c-KIT kinase inhibitor, Seralutinib, in PCLS from Group 1 PH patients [94]. Ex vivo treatment decreased the established pulmonary artery muscularization (αSMA), and increased apoptosis (TUNEL), with this data supporting the progression of Seralutinib to phase III trials as an inhaled formulation for PAH (PROSERA; NCT05934526). This provides a clear example of the how ex vivo assessment of the effects of treatments on the complex interplay between disease-relevant readouts in PCLS could contribute to rapid clinical translation.

#### Molecular signatures

Methods to isolate quality RNA yields from agarose-inflated PCLS from mouse, pig, and human lung have now been established [95]. Furthermore, RNA sequencing has been performed on single-cell suspensions from human PCLS to great effect, allowing for the identification of cell-specific signatures in IPF lung samples [92] and the effects of e-cigarette extract treatment in combination with influenza A infection ex vivo [9]. These approaches can now be applied to PCLS treated ex vivo to mimic disease conditions or to PCLS prepared from animal models of PH or from human subjects. This will facilitate greater insights into disease-specific molecular signatures and mechanisms underlying responses such as altered reactivity and vascular remodelling.

## Conclusion

As noted in a recent ATS Workshop report [8], PCLS from healthy and diseased tissue provide a unique tool which can 1) recapitulate the complexity of the lung’s native environment, 2) enable the study of the complex interactions among different cell types and the extracellular matrix (ECM) in the lungs’ native 3D architecture, 3) allow for high resolution (live) imaging of cellular functions in several dimensions, 4) mimic the onset and progression of lung diseases, complementing studies in end-stage diseased tissue, and 5) facilitate the testing of potential therapeutics in disease-relevant models.

**Fig. 3:**
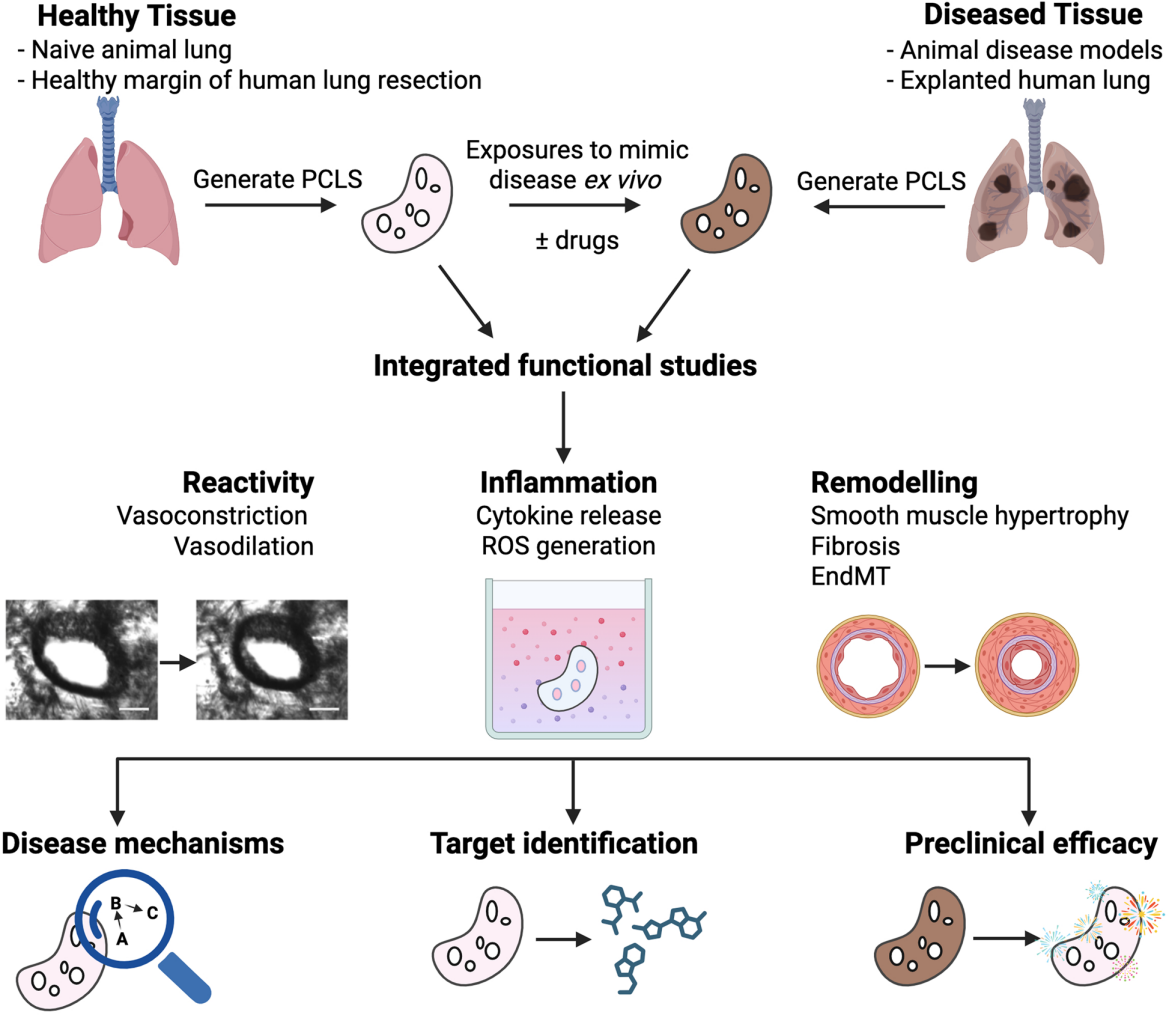
Overview of integrated functional studies using animal or human precision-cut lung slices for investigating mechanisms and therapeutics for pulmonary hypertension. Multiple PCLS with preserved pulmonary arteries can be generated either from healthy tissue, or tissues taken from animal models of disease, or patients with the disease of interest. Where diseased patient tissue is unavailable, mediators that mimic the disease milieu can be administered ex vivo to essentially create “diseased” tissue for comparison. This then allows for comprehensive studies of vascular reactivity integrated with inflammatory responses and tissue remodelling in the lung, with the potential to investigate multiple readouts in a single PCLS. Overall, the use of animal and human PCLS in this way allows for elucidation of disease mechanisms, identification of therapeutic targets, and assessment of preclinical efficacy, which may all contribute to clinical translation of research. Made with Biorender

PCLS can be used for assessment of disease-relevant readouts including reactivity, inflammation and remodelling in situ in multicellular PCLS preparations (Fig. 3), offering significant advantages relative to cell-based studies. Additionally, the capacity to prepare multiple PCLS from a given lung sample, including those from resected or explanted human lungs, provides higher throughput and addresses ethical considerations compared to labour-intensive in vivo studies in animal models.

Through the application of PCLS in the context of pulmonary hypertension, mechanistic insights extending from physiology to pathophysiology have been gained, with PCLS also providing a vital platform for therapeutic evaluation. Further studies are required to extend and translate this knowledge, using PCLS to identify new strategies to address the limitations of current treatments and improve patient outcomes.

## Data Availability

The datasets used and/or analysed during this study are available from the corresponding author upon reasonable request.

## Abbreviations

5-HT: 5-Hydroxytryptamine (serotonin)
ACh: Acetylcholine
ARDS: Acute respiratory distress syndrome
BPD: Bronchopulmonary dysplasia
CD: Cluster of differentiation
COPD: Chronic obstructive pulmonary disease
DMEM: Dulbecco’s modified eagle medium
DMSO: Dimethyl sulfoxide
ECM: Extracellular matrix
EndMT: Endothelial-mesenchymal transition
ET-1: Endothelin-1
FC: Fibrotic cocktail
FPR: Formyl peptide receptor
GABA-R: Gamma-aminobutyric acid receptor
GM-CSF: Granulocyte macrophage-colony stimulating factor
IL: Interleukin
IPF: Idiopathic pulmonary fibrosis
JAK2: Janus kinase 2
LPA: Lysophosphatidic acid
LPS: Lipopolysaccharide
MCP-1: Monocyte chemoattractant protein-1
NO: Nitric oxide
NOC-5: 3-[2-Hydroxy-1-(1-methylethyl)-2-nitrosohydrazinyl]-1-propanamine
OVA-HX: Ovalbumin-hypoxia model of PH
PA: Pulmonary artery
PAH: Pulmonary hypertension
PAP: Pulmonary arterial pressure
PCLS: Precision-cut lung slice
PDE: Phosphodiesterase
PDGF: Platelet-derived growth factor
PH: Pulmonary hypertension
RNA: Ribonucleic acid
TGF-β: Transforming growth factor-β
TKI: Tyrosine kinase inhibitor
TNF-α: Tumor necrosis factor-α
